# Formulation and In Vivo Evaluation of Biofilm Loaded with Silver Sulfadiazine for Burn Healing

**DOI:** 10.3390/gels9110855

**Published:** 2023-10-29

**Authors:** Doaa Alshora, Lubna Ashri, Rihaf Alfaraj, Ahlam Alhusaini, Raeesa Mohammad, Nawal Alanaze, Mohamed Ibrahim, Mohamed M. Badran, Mounir Bekhit, Shaikha Alsaif, Modhi Alagili, Rehab A. Ali, Adel Jreebi

**Affiliations:** 1 Department of Pharmaceutics, College of Pharmacy, King Saud University, P.O. Box 22452, Riyadh 11459, Saudi Arabia; lashri@ksu.edu.sa (L.A.); ralfaraj@ksu.edu.sa (R.A.); nalanaze@ksu.edu.sa (N.A.); mhamoudah@ksu.edu.sa (M.I.); mbadran@ksu.edu.sa (M.M.B.); mbekhet@ksu.edu.sa (M.B.); modi-modi-modi@hotmail.com (M.A.); ajriby@ksu.edu.sa (A.J.); 2 Department of Pharmacology and Toxicology, College of Pharmacy, King Saud University, P.O. Box 22452, Riyadh 11459, Saudi Arabia; aelhusaini@ksu.edu.sa (A.A.); reali@ksu.edu.sa (R.A.A.); 3 Department of Histology, College of Medicine, King Saud University, P.O. Box 2925, Riyadh 11461, Saudi Arabia; rmohammad@ksu.edu.sa

**Keywords:** silver sulfadiazine, biofilm, release study, antibacterial activity, burn wound model

## Abstract

Infected burned skin is a life-threatening condition, which may lead to sepsis. The aims of this work are to formulate a biofilm composed of silver sulfadiazine (SSD), chitosan (CS), and sodium alginate (SA), and to evaluate its wound-healing effectiveness. A full factorial design was used to formulate different matrix formulations. The prepared biofilm was tested for physicochemical, and in vitro release. The optimized formulation is composed of 0.833% of CS and 0.75% of SA. The release of SSD almost reached 100% after 6 h. The mechanical properties of the optimized formula were reasonable. The antibacterial activity for the optimized biofilm was significantly higher than that of blank biofilm, which is composed of CS and SA, *p* = 1.53922 × 10^−12^. Moreover, the in vivo study showed a 75% reduction in wound width when using the formulated SSD biofilm compared to standard marketed cream (57%) and the untreated group (0%).

## 1. Introduction

Burns can be either superficial or deep enough to reach and damage the epidermis and dermis in the subcutaneous tissue. The primary causes of burns are thermal; however, they may be caused by extensive exposure to sunlight, radiation, chemicals, and electricity. According to the World Health Organization [1], burns are a global health problem, and burn sepsis is the primary cause of death in patients with burns [1,2].

Burns and wounds are susceptible to infection by bacteria, including *Escherichia coli*, *Pseudomonas aeruginosa*, *Enterobacter* species, Actinobacter species, Proteus species; *Klebsiella* species as gram-negative bacteria, and *Staphylococcus aureus*; *Enterococcus* species as gram-positive bacteria [3]. Therefore, the use of topical antibacterial agents is vital for preventing wound and burn infections.

Silver sulfadiazine (SSD) is a sulfonamide product with broad-spectrum antibacterial activity against most common gram-positive and gram-negative species, as well as against yeast. Although SSD (1%) is the first-line treatment for burn injuries, it has some drawbacks. The cream dries with time and needs frequent application. In some cases, covering the burned area with gauze is necessary, and the procedure of applying gauze could be painful for the patient, since the gauze could adhere to tissue, making the removal process painful [4].

Several clinical comparative studies have been performed to evaluate the effectiveness of SSD compared to other burn treatments. Homann et al. compared the effect of SSD with that of a liposome hydrogel containing polyvinylpyrrolidone iodine. The results showed complete healing within a short time following treatment with the liposomal formulation (10 days), compared to approximately 12 days following treatment with SSD [5]. Huang et al. compared the effects of SSD and Acticoat dressings. Acticoat is a silver-impregnated antimicrobial barrier dressing that can be used for 3 days. The results revealed that the healing time decreased from 15.79 days with SSD to 12.42 days with Acticoat dressing [6].

SSD has poor water solubility and limited solubility in most organic solvents. Several studies have been conducted to formulate SSD film with chitosan as a composite and the complete release of the drug from the film was found to take days [3,7,8]. Therefore, the formulation of biodegradable films containing burn-treatment drugs, such as SSD, could overcome these drawbacks. Chitosan and alginate are the most commonly used natural products for biodegradable film preparation. Chitosan is widely used in medicine because of its low toxicity, biocompatibility, and biodegradability. Moreover, chitosan has antimicrobial effects and is used as a wound-healing accelerator [9,10]. The antibacterial activity of chitosan is controlled by many factors, including the molecular weight, and degree of deacetylation, as well as other active ingredients. The antibacterial activity is proportionally related to the degree of deacetylation and indirectly to the molecular weight [11,12]. The addition of silver nanoparticles enhances and promotes wound healing and tissue regeneration [11]. Alginate, a natural polysaccharide, acts as a biocompatible hydrophilic gel. This gel has the advantage of regulating a moist environment and promoting the healing and regeneration of epidermal tissue [13]. In addition, it decreases inflammatory reactions and enhances type I synthesis [14,15,16]. Several attempts were conducted to incorporate SSD, chitosan, and alginate in dosage forms, El-Feky et al. [17] formulated alginate-coated chitosan nanogel. The in vitro dissolution study showed that only 50% of the drug was released within 3 h. Moreover, the percentage of wound reduction was 49%. Kim et al. [18] incorporated SSD in a chitosan sponge. The in vivo study showed that complete wound-healing took 4 weeks.

However, burns are life-threatening because they attenuate the immune system, thus reducing the ability of the body to prevent infection. Therefore, controlling infection to the greatest possible extent is crucial. The aim of the present experimental work was to formulate a biofilm containing a soluble SSD with dual action. Firstly, SSD can control infection. Secondly, the prepared biofilm, which is composed of chitosan and alginate, also has antibacterial activity and can act as a healing agent [19,20].

## 2. Results and Discussion

### 2.1. Experimental Design

This study aimed to formulate SSD biofilms containing chitosan and sodium alginate. The effects of different concentrations of chitosan and sodium alginate on mechanical properties and drug release were studied.

#### 2.1.1. The Effect on the Mechanical Properties of Dermal Films Containing SSD

The evaluation of the mechanical properties of dermal films containing SSD is essential for assessing the physical integrity of the film [21]. Two mechanical properties of the prepared SSD dermal films were evaluated: tensile strength and elongation at break (%EB). Tensile strength (TS) refers to the maximum stress per cross-sectional area that the film can withstand before breaking and provides a sign of film strength. The %EB evaluates the ability of the film to stretch before breaking, as it describes film elasticity. %EB is the percentage of the change in film length [22].

**Figure 1:**
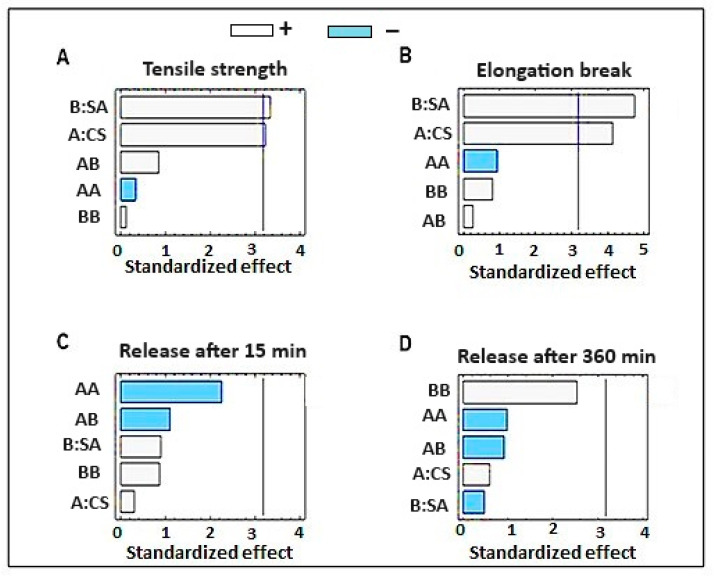
Pareto chart for the effect of different concentrations of sodium alginate and chitosan on (**A**) tensile strength, (**B**) elongation break, (**C**) release after 15 min, and (**D**) release after 360 min.

**Figure 2:**
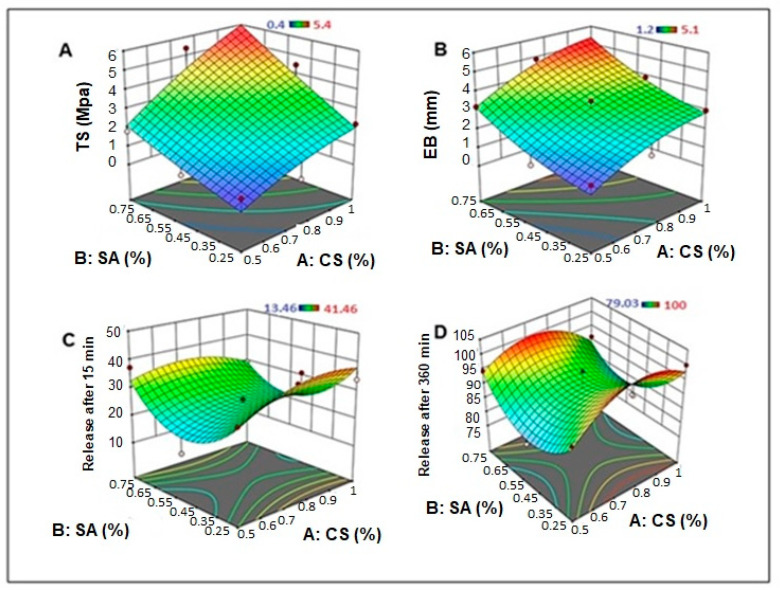
3D response surface plot for the independent factors on (**A**) tensile strength, (**B**) elongation break, (**C**) release after 15 min, and (**D**) release after 360 min.

An ANOVA analysis of the effects of the independent variables on the dermal film tensile strength, TS (Y1), is displayed in Table 1. The film constituents CS and SA exhibited significant agonistic effects on the film tensile strength (*p* = 0.0481 and 0.0422, respectively), whereas the interactive effects (AB) and quadratic effects of SA concentration (BB) showed an agonistic but insignificant effect, as shown in the Pareto-standardized chart (Figure 1A). Moreover, the quadratic effect of the CS concentration showed an antagonistic but had an insignificant impact on the film TS. The 3D response surface plot (Figure 2A) indicated that increasing the concentrations of CS and SA increased the TS of the film, where the highest value of film TS (5.2 and 5.4 Mpa) was recorded for the film formula containing the highest level of SA, with the highest and medium levels of CS (F2 and F4, respectively) (Table 2).

Similarly, CS and SA had significant agonistic effects on film extension at break load: %EB (Y2), where the recorded *p*-values were 0.0257 and 0.0179, respectively, as shown in the ANOVA results (Table 1) and the Pareto standardized chart (Figure 1B). In addition, the interactive effects (AB) and quadratic effects of the SA concentration (BB) had an agonistic but insignificant impact, and the quadratic effect of the CS concentration had an antagonistic but insignificant effect on the EB of the dermal film. As shown in the 3D response surface plot (Figure 2B), the highest values of dermal film %EB (5.1 ± 0.483 and 4.9 ± 0.410 mm) were recorded for film formulations F4 and F2, respectively (Table 2), which contain the highest and moderate concentrations of CS, respectively, with the highest concentration of SA. In addition, a low EB was observed for the films containing low levels of CS and SA (F6 and F9).

As mentioned previously, the highest film strength, along with the highest flexibility (indicated by %EB), was recorded in cases where concentrations of both CS and SA were high. This can be attributed to the role of the cationic (chitosan (CS)) and anionic (sodium alginate (SA)) additives in improving the mechanical properties of the film, including membrane flexibility and resistance [23]. This may be due to the formation of polyelectrolyte complexes (PEC) between CS and SA. Yan et al. [24], showed that CS-AL PEC films have remarkable tensile properties that are dependent on the polymer concentration. Moreover, crosslinking between CS and SA can reduce the hydrophilicity of the polymer and improve its mechanical and thermal properties [25].

#### 2.1.2. The Effect on the In Vitro SSD Release from Dermal Films

The effects of independent parameters (CS and SA) on the initial in vitro drug release (after 30 min) and final drug release are displayed in ANOVA Table 1 and Pareto-standardized charts (Figure 1C,D). The ANOVA showed that neither the individual parameters (CS and SA) nor their interactive or quadratic effects had any significant effect on the drug release rate from the dermal films (after 30 min and 360 min), where the calculated *p*-values were >0.05 for all effects. The quadratic effects of CS and SA had antagonistic but insignificant effects on the initial and final in vitro drug release, whereas the individual and interactive effects showed agonistic but insignificant effects on the response.

The 3D response surface plots (Figure 2C,D) revealed that increasing the concentration of CS resulted in a slight increase in the in vitro drug release from the dermal film, and a decrease in the in vitro release rate was observed for the highest CS levels. In contrast, increasing the concentration of SA in the dermal film resulted in a slight decrease in the initial and final in vitro drug release rates, and an increase in the drug release rate was observed at higher SA levels.

**Figure 3:**
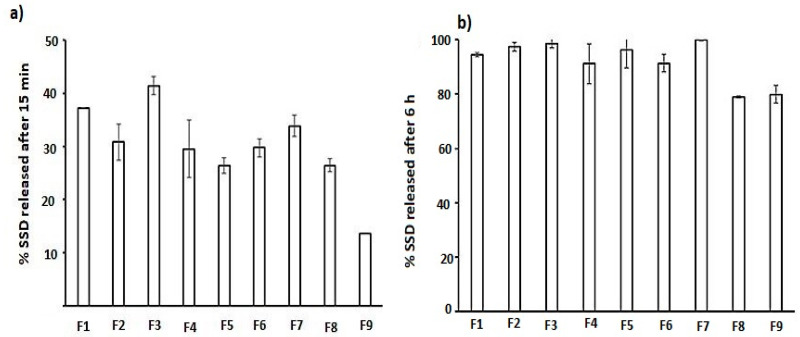
The release percentage of SSD after (**a**) 15 min and (**b**) 6 h.

**Figure 4:**
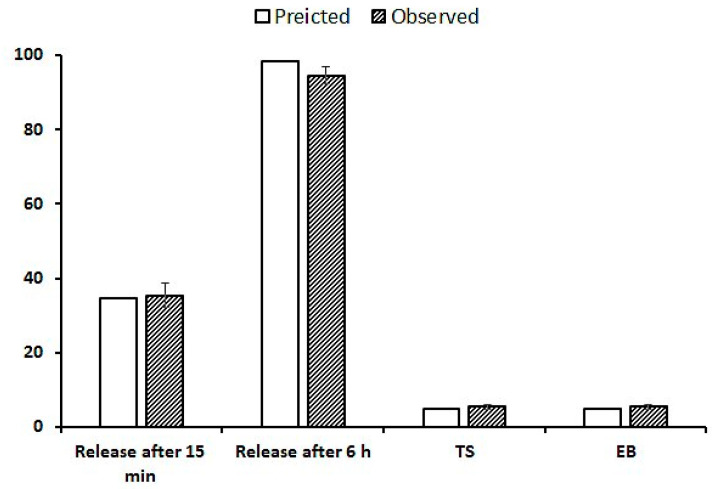
Properties of the optimized formulation.

Figure 3 (histogram) shows the release of SSD from the biofilm after 15 min. F3, F6, and F7 showed the highest release, and these formulations contained a low concentration of SA, which was not sufficient to form a firm film. Therefore, the disruption of the film structure occurred once it was placed in the dissolution medium, leading to the release of 100% of the drug after 6 h (Figure 4) [26].

The release behavior from F1, F2, and F4, which contain the highest SA concentration (0.75%), shows that the increasing polymer-to-drug ratio (CS + SA: drug) significantly decreases the release from 37.3 ± 0 (F1) to 29.56 ± 5.53 in F4 after 15 min (Figure 3a) and from 94.5 ± 0.79 to 91.27 ± 5.54 after 6 h for F1 and F4, respectively (Figure 3b). This retardation could be due to an increase in the polymer ratio, which forms a thick gelatin layer that controls access to the dissolution media. In other studies, the crosslinking between SA at a concentration of 0.75% and different concentrations of CS was high, and a firm film structure was produced that could retard the release of SSD [27].

### 2.2. Swelling Index Study

The swelling of the gel matrix provides an opportunity for the solvent to enter the matrix through the channel created due to the relaxation of the polymer chain. This may also depend on the polymer–drug ratio; increasing the polymer–drug ratio may provide a rigid matrix that takes time to swell so that less of the drug is released [17]. The data of the swelling index were correlated with the release study. The results revealed that, at a constant CS concentration, increasing the SA concentration decreased the swelling index, and thus a low drug release was observed (Table 2). Formulations F7 and F8 are composed of 1% CS and an SA of 0.25 and 0.5%, respectively. The swelling index decreased from 3.9 to 2.9%, which reflects a correlated release reduction from 33.9 to 26.5%. The formulation prepared with 0.5% CS acted in the same behavior; the swelling index decreased from 3.5 in F6 (0.25% SA) to 3.2 in F9 (0.5% SA). This reduction affects the drug release, which decreased from 29.8 (F6) to 13.5 in F9.

The results showed that drug release from the biofilm formulations is controlled by the participation of parameters based on the CS and SA levels in the formula, as well as their interactions. These parameters affected the drug swelling properties as well as the tensile strength of the biofilm. For example, a rapid initial drug release (F1, F3, and F7) was found in the biofilm formulations with high swelling indices. However, other formulations that exhibited high swelling indices (F2, F4, and F8) showed slower initial drug release rates, and these formulations showed increased tensile strength values.

### 2.3. Release Kinetics

Table 3 shows the release kinetics of SSD from different biofilm formulations. The release data for the zero-order, first-order, Higuchi, and Korsmeyer–Peppas models were plotted. The fitting of the release kinetics was determined using the highest correlation coefficient (r).

Release from the biodegradable polymer may occur by diffusion of the solvent through the crosslinked system. Swelling occurs followed by the dissolution of macro-molecules. The release kinetics data showed that all formulations fit the Higuchi model. The n value from the Korsmeyer–Peppas model was <0.45, indicating a nonfiction diffusion mechanism [17,28,29].

### 2.4. Optimization of Formulation Parameters

Optimization was based on certain desirability, including the maximum mechanical properties and maximum release after 15 and 360 min. The program suggests a formulation composed of 0.833% of CS and 0.75% of SA. The optimized formulation was characterized using X-ray diffraction, DSC, and mechanical and release characteristics. Figure 4 shows a comparison of the predicted and observed values. After 15 min of dissolution, 35.4% of SSD was released, compared with 34.58% of the observed value. At the end of the dissolution run, 94.6% of SDD was released, compared to 98.21% of the observed value.

### 2.5. Differential Scanning Calorimetry (DSC)

**Figure 5:**
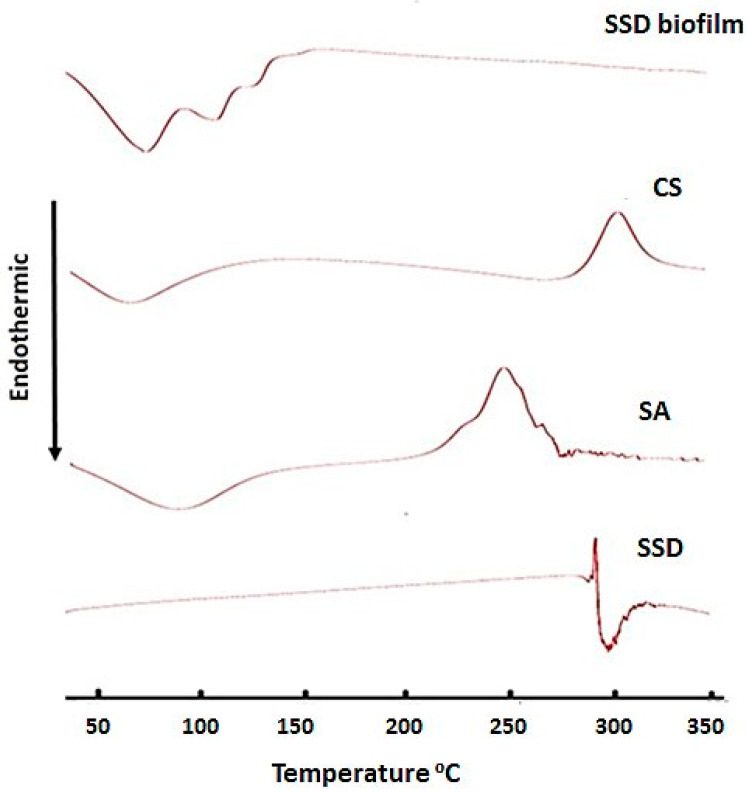
The DSC scan for SSD, CS, SA, and SSD film.

DSC thermos analysis for SSD-, CS-, SA-, and SSD-loaded biofilms was performed, and the results are shown in Figure 5. DSC analysis of SSD showed an exothermic peak at 288 °C, which could be related to the drug decomposition, and an endothermic peak at 290 °C, indicating the melting and dehydration of the drug [8]. CS shows an endothermic peak at 65.9 °C, which corresponds to dehydration temperature, while the exothermic peak that appears at 307 °C is related to chitosan decomposition. In addition, SA showed both endothermic and exothermic peaks at 85.7 and 250 °C, respectively. The thermal analysis for the film shows an endothermic peak at 72.3 °C, which may be associated with the dehydration and melting of CS and SA, with the absence of an SSD peak. The disappearance of the drug-melting endotherm might denote the homogeneous dispersal of SSD in the matrix of the CS-SA film [30], as well as the loss of its crystallinity, which could cause regular drug release from the film matrix.

### 2.6. X-ray Powder Diffraction (XRPD)

**Figure 6:**
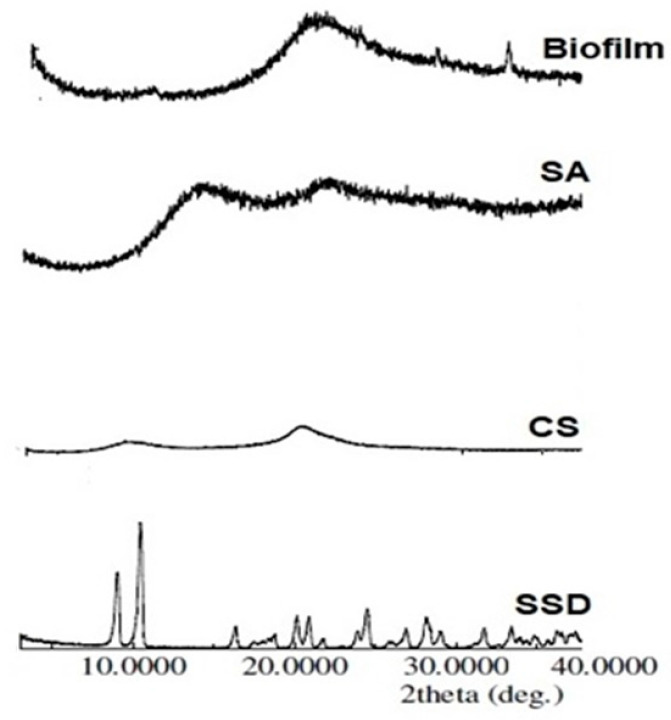
The XRPD spectra of SSD, CS, SA, and SSD film.

The X-ray powder diffraction patterns of the SSD-loaded biofilm compared with film constituents are displayed in Figure 6. The diffraction spectrum of SSD showed several diffraction peaks at diffraction angles of 2θ degree at 9.04A°, 10.48A°, 20.14A°, 20.880A° and 24.48A°, while the intensities of these diffraction peaks were 518, 896, 202, 211 and 256, respectively. In contrast, both CS and SA did not show distinct diffraction peaks. The XRPD spectrum of SSD-loaded biofilm showed the disappearance of SSD characteristics’ diffraction peaks, representing the loss of its crystallinity, along with homogeneous dispersion of the matrix of the biofilm [31].

### 2.7. Analysis of the Antibacterial Activity of SDD Film

#### 2.7.1. Analysis of the Antibacterial Activity of SSD Film Using Agar Diffusion

**Figure 7:**
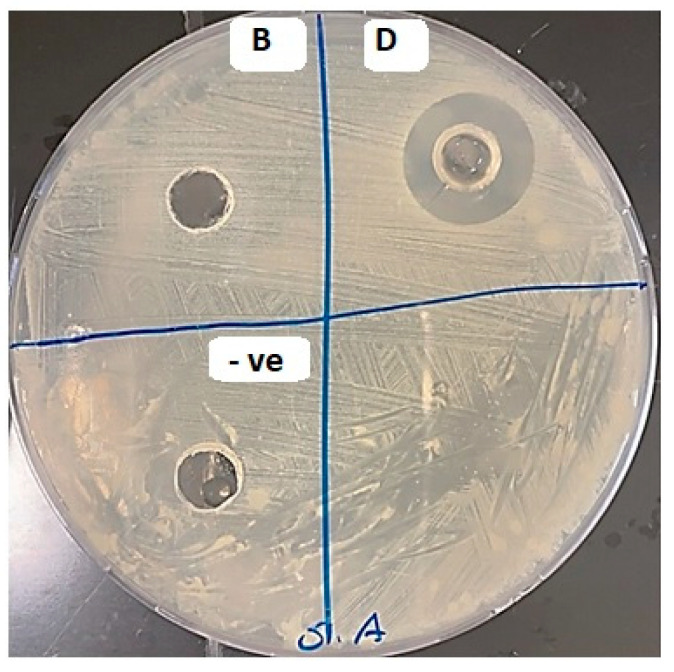
The antibacterial effect of SSD film compared with non-medicated film on different zone in the agar plate, zone B for the non-medicated film and zone D for the medicated film (SSD biofilm).

The agar well diffusion demonstrated in Figure 7 has an inhibitory effect on different zones: zone B for the non-medicated film and zone D for the medicated film (SSD film). A zone of inhibition around the well filled with SSD film was obvious compared with the non-visible inhibition zone around the non-medicated film, indicating weak antibacterial activity in the non-medicated film. Therefore, the antibacterial activity was analyzed in liquid culture to ensure the activity of the non-medicated film.

#### 2.7.2. Analysis of the Antibacterial Activity of SSD Film in Liquid Culture

**Figure 8:**
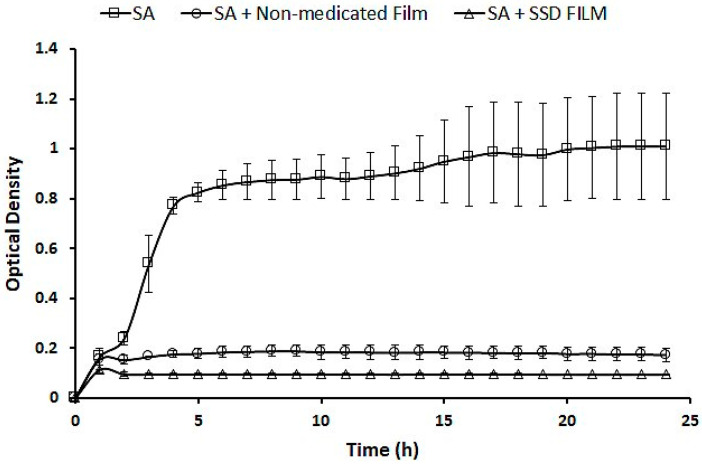
Analysis of the antibacterial activity of SSD film in liquid culture.

The effect of the SSD film was compared to that of a non-medicated film using *S. aureus*. Figure 8 illustrates the antibacterial activity of the SSD film compared to that of the non-medicated film. The data show a significant reduction in SA growth in film products. The non-medicated film significantly reduced the growth of SA, with a *p*-value of 2.08655 × 10^−15^. The bacterial reduction was also extremely significant for the film loaded with SSD (*p* = 2.42016 × 10^−17^). These results demonstrated the antibacterial activity of the non-medicated film. However, the SSD film showed a significant reduction in bacterial growth (*p* = 1.53922 × 10^−12^).

### 2.8. Burn Wound Healing Measurement

**Figure 9:**
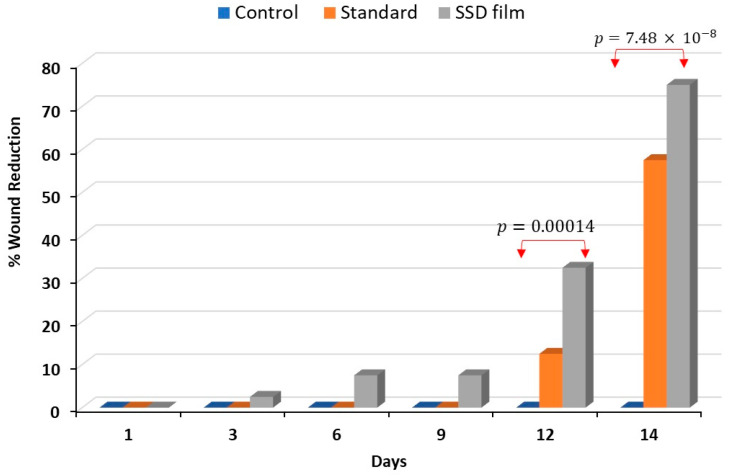
The percentage of burn wound healing after application of SSD biofilm compared with the untreated group and group treated with standard marketed cream.

The percentage of wound reduction was calculated on days 3, 6, 9, 12, and 14. Figure 9 shows that no reduction in the wound width occurred over 14 days in the control (untreated) group. Group II, which was treated with marketed SSD cream, began to display a reduction in burn wound width on the 12th day of treatment and reached 57% on day 14. Interestingly, the reduction in the wound width in rats treated with SSD film (Group III) started on the third day. The percentage of wound reduction increased during the treatment period from 2.5% on the third day to reach 75% on day 14.

### 2.9. Histopathological Analysis

**Figure 10:**
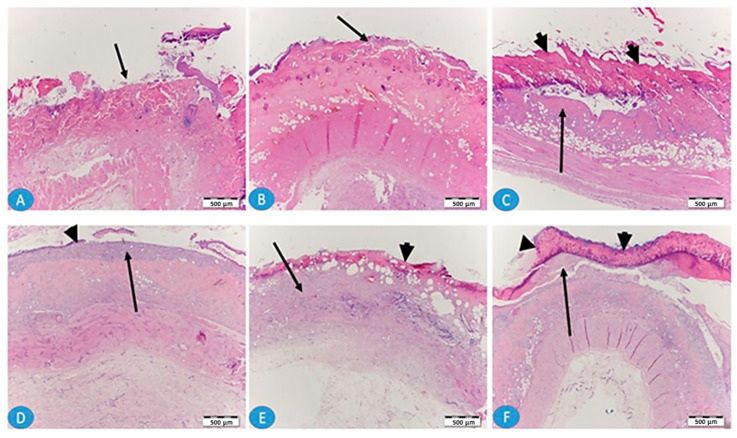
Photomicrographs of H&E-stained skin sections after one week (**A**–**C**) and after 2 weeks (**D**–**F**) of local burning. (**A**,**D**) The untreated group; (**B**,**E**) the group treated with marketed SSD cream; (**C**,**F**) the group treated with SSD biofilm.

The histopathological examination was carried out on days 7 and 14 to assess any morphological changes that occurred during the treatment period in the three groups. Figure 10 shows skin sections after one week (A, B, and C) and two weeks (D, E, and F) of local burning. Photos A and D reflect the untreated skin sections in which there is a clear, bare wound without epithelium covering (arrows). After one week, there was no granulation tissue, while after two weeks there was prominent granulation tissue filling the wound (D, arrow) and a small thin epithelium tissue (D, arrowhead). Photos B and E from skin treated with local application of standard marketed SSD cream, show that, after one week (B), more granulation tissue is apparent while epithelization remained poor (arrow). Photos C and F from skin treated with SSD biofilm show that, after one week (C) and 2 weeks (F), the epithelization was more enhanced, covering almost all the surface of the wound (arrowheads), and more mature epithelium was observed after 2 weeks of application of the SSD biofilm.

**Figure 11:**
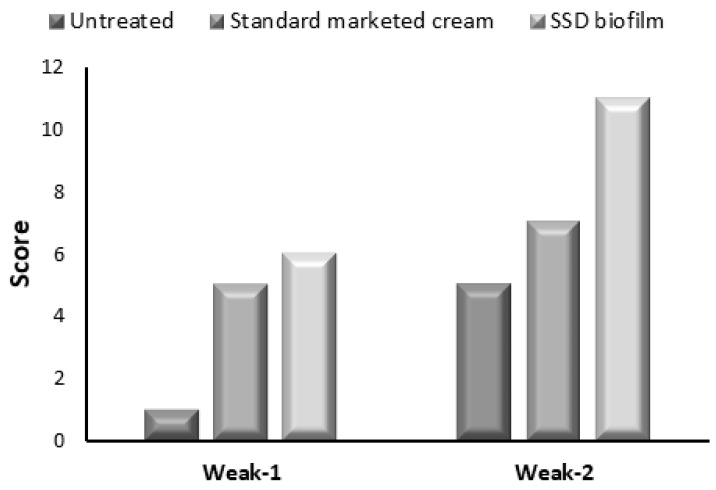
Histology scoring for burned area healing in the three groups.

Figure 11 shows the histological score of the burned area according to Greenhalgh et al.’s criteria [32]. In the first week, the burned skin obtained from the untreated group exhibited a score of 1, which indicates that there is no granulation tissue without epithelial formation, while in the second week, the resulting score indicates thin and immature granulation with minimal epithelial migration. In the group using SSD cream, the histological score of the burned area indicates immature granulation dominated by inflammatory cells in the first week. In the second week, the specimen of this group scored 7, which indicates moderately thick granulation tissue and moderate epithelium migration. Interestingly, the specimen obtained from the group using the SSD biofilm exhibited a score of 11 in the second week, which indicates a thick vascular granulation tissue dominated by fibroblast, extensive collagen deposition, and epithelium that completely covers the wound.

## 3. Conclusions

Globally, burns are problematic health issues. Burn infections, which could be exaggerated to sepsis, are considered the number one cause of death. The formulation of SSD biofilm showed promising results in controlling the infection and recovering the burned skin. Although the antibacterial activity of non-medicated (CS-SA) film showed a significant reduction in bacterial growth, the data also demonstrate the significance of the formulated SSD biofilm. Formulating a dosage form that can deliver SSD and decrease the infection with *Staphylococcus aureus* is an extremely useful intervention in the treatment of the infected burn. The histology of the wound area showed that epithelization starts after 1 week of treatment and covers the wound area with extensive collagen deposition after 2 weeks.

## 4. Materials and Methods

### 4.1. Materials

SSD was obtained from Riyadh Pharma Pharmaceutical Company, (Riyadh, Saudi Arabia). Medium-molecular weight (MMW) chitosan (deacetylation degree of ≥75%) was purchased from Sigma-Aldrich (Schnelldorf, Germany). Glacial acetic acid was obtained from BDH (Leicestershire, UK). All the remaining chemicals were of analytical grade.

### 4.2. Experimental Design

The impact of different concentrations of chitosan (X1; 1, 0.75, 0.5%) and sodium alginate (X2; 0.75, 0.5, 0.25%) was studied using response surface methodology at three levels (32) with the aid of Statgraphics software (version 17.2.02.; Statgraphics Centurion). The influence of these factors on several parameters, including tensile strength (Y1) and in vitro release (Y2), was studied. Table 4 shows the variable levels and different formulation compositions.

### 4.3. Preparation of Film Loaded with SSD

Biofilms were prepared using the solvent casting method. Chitosan was dissolved separately in 1.5% acetic acid. SSD (1%) was dissolved in a certain amount of water containing 1 mL of ammonia solution (32%) and 5% of propylene glycol. After the complete dissolving of the drug, a calculated amount of sodium alginate was added. The chitosan solution was then added to the alginate mixture and a homogenous viscous mixture was prepared using a homogenizer. The mixture was poured into a round casting mold with a diameter of 85 mm. The solvent was then evaporated in an oven at a fixed temperature of 40 °C for 12 h. The dried films were then stored in a desiccator and protected from light until further investigation.

### 4.4. Mechanical Properties

The mechanical properties of the prepared biofilms were evaluated by measuring the tensile strength (TS) and elongation at break (EB). The test was performed at room temperature using an Instron universal testing machine (model 8500 digital control, Instron) equipped with a loading cell of 5 kN. Initially, the biofilms were cut into 1 × 3 cm strips; their thicknesses were measured using an electronic digital caliper (Ultra-Cal Mark III, Fred V. Fowler Co., Newton, MA, USA), and the average values were recorded (n = 2). Each biofilm strip was then fixed between the two grip fixtures of the machine, and the upper grip was withdrawn at a crosshead speed of 20 mm/min. When the biofilm broke, force and elongation were measured twice for each batch. Finally, dedicated software (Instron series IX, version 8.32.00) was used to analyze the resulting profiles and calculate the tensile strength (TS), which was expressed in MPa (N/mm^2^). Extensibility, which is the elongation to break, was expressed as a percentage and calculated using the following formula:

$$Extensibility=\frac{increase\;in\;length}{\;initial\;length}\times 100$$

### 4.5. Assessment of Swelling

The swelling behavior of each of the nine biofilm formulations was studied at pH 7.4. A dry biofilm weighing 100 mg was placed in a basket and immersed in 20 mL buffer pH 7.4 warmed and adjusted to 37 °C. After a certain time interval, and for 2 h, the basket was removed and placed on filter paper to remove excess water. The swelling index was calculated using the following equation:

$$SI=\frac{W_{w}-W_{o}}{W_{o}}$$

where *W_o_* is the weight of the biofilm in the dry state, and *W_w_* is the weight of the wet biofilm after a certain time.

### 4.6. In Vitro Drug Release

The drug release was performed using a dissolution apparatus II fixed at a rotation speed of 75 rpm. The test was carried out in a phosphate buffer saline pH 7.4, adjusted at a temperature of 37 ± 0.5 °C. A sample of 5mL was withdrawn for 6 h. The sample was spectrophotometrically measured at λ_max_ of 254 nm.

### 4.7. Release Kinetics

Different kinetic models were used to analyze the in vitro release data. The release data obtained for various SSD biofilms were fitted to the zero-order, first-order, Higuchi model, and Korsmeyer–Peppas model. The n value from the Korsmeyer–Peppas model was calculated to investigate the release mechanism.

### 4.8. Differential Scanning Calorimetry (DSC)

The thermal behaviors of the SSD, Cs, SA, and SSD biofilms were evaluated using differential scanning calorimetry (DSC 8000 Perkins Elmer, Waltham, MA, USA) apparatus within the temperature range of 25–350 °C at a heating rate of 10 °C/min. Pyris management software version 10.1 (Pyris Elmer, Waltham, MA, USA) was utilized to assess the samples.

### 4.9. X-ray Powder Diffraction

The crystallinity nature of the drug was then evaluated. XRPD scans were acquired for the optimized SSD biofilms, compared with the individual ingredients using a RIGAKU diffractometer (Rigaku Corporation, Tokyo, Japan), and equipped with a curved graphite crystal monochromator, automatic divergence slit, and automatic controller (PW/1710). Cu Kα radiation working at 40 KV and 40 mA (λkα = 1.5418 Å) was used as the target. The diffraction measures were achieved using continuous scan mode with a 2ϴ degree range from 4° to 60°.

### 4.10. Antibacterial Activity

#### 4.10.1. Analysis of the Antibacterial Activity of SSD Film in Liquid Culture

*S. aureus* was grown in 5 mL of Luria–Bertani (LB) broth before being incubated at 37 °C with continuous shaking at 200 rpm for 16 h. Then, 200 µL of bacterial broth with an optical density of 0.5 McFarland was added to a 100-well plate, after which the bacterial culture was divided into three samples: non-treated *S. aureus*, *S. aureus* treated with non-medicated film, and S. aureus treated with SSD film. Finally, the 100-well plate was placed in a Bioscreen C reader, and the optical density of bacterial growth was recorded at 600 nm for 24 h.

#### 4.10.2. Analysis of the Antibacterial Activity of SSD Film Using Agar Diffusion

An *S. aureus* was grown in 5 mL of LB broth before being incubated at 37 °C with shaking at 200 rpm for 16 h. Then, 200 µL of bacterial broth with an optical density of 0.5 McFarland was added to the Petri dishes using the spread plate method, and a 6 mm well was cut in the middle of each plate. Next, 100 µL of the negative control, SSD film, and non-medicated film were added to the well. After 16 h of incubation at 37 °C, the plates were examined for growth inhibition surrounding the well.

### 4.11. In Vivo Burn Healing Activity

#### 4.11.1. Animals

Eighteen adult male albino rats weighing 200–250 g were obtained from the Experimental Animal Center, King Saud University. The animals were allowed to adapt in the laboratory for one week under standard environmental conditions of temperature 22 °C and natural light/dark cycle. They were given a standard rat pellet diet and distilled water ad libitum. The use of animals and experimental design were approved by the Scientific Research Ethics Committee, King Saud University [KSU-SE-23-19].

#### 4.11.2. Experimental Design

The animals were housed in individual cages with ad libitum access to water and food (rodent chow). Anesthesia was implemented by i.p. injection with thiopental (20 mg/kg) with ketamine (20 mg/kg). The dorsal hair of the area proposed to be burned was shaved and left for 24 h before infliction to minimize local inflammation. Then, the burn was inflicted by holding a 1.5 cm^2^ aluminum square, previously heated at 100 °C, over the exposed skin for 5 s. Haste samples weighing 200 g were connected to an aluminum square before infliction. Therefore, the pressure applied to the skin (owing to the weight of the haste) was the same for all animals. To treat postoperative pain, 10 mg/kg tenoxicam was administered intramuscularly to the rats once daily for three days. The animals were divided into three groups: group I were untreated, group II was treated with SSD-marketed cream, and group III was treated with SSD biofilm. Medicated SSD biofilm was cut to the same wound size and placed over the burned area. The rats were observed regularly until the wounds were completely healed. A burn wound model was used to evaluate the rate of wound contraction and time required for full epithelialization of the wound. The percentage of wound closure was calculated as follows:

$$\%\;of\;wound\;reduction=\frac{A_{0\;}-A_{t}}{A_{0}}\times 100$$

where *A*_0_ is the original wound area and *A_t_* is the area of the wounds evaluated every three days; that is, on days 3, 6, 9, 12, 15, and 18 [33].

After the completion of the experiment, three rats were sacrificed on the 7th day, and the other three on the 14th day using CO_2_. The skin specimens were fixed in 10% formalin for histological examination.

### 4.12. Histopathological Analysis

Then, 5 μm sections from the mid-portion of the wound were stained by Hematoxylin and Eosin (H&E). Each slide was given a histological score ranging from 1 to 12 (Table 5), with 1 associated with no healing and 12 associated with a completely re-epithelialized wound [32].

### 4.13. Statistical Analysis

Data were expressed as mean ± SEM and the statistical comparisons were performed using the one-way analysis of variance (ANOVA), followed by the Tukey–Kramer multiple comparisons post hoc test. Statistical significance was set at *p* < 0.05.

## Figures and Tables

**Table 1:** ANOVA for the effects of independent parameters of the properties of SSD-loaded dermal films.

Tensile Strength (Mpa); Y1	**Source**	**Sum of Squares**	**F-Ratio**	***p*-Value**
	A-CS	12.91	10.46	0.0481
	B-SA	13.80	11.18	0.0442
	AB	0.9025	0.7314	0.4553
	A^2^	0.1422	0.1153	0.7566
	B^2^	0.0139	0.0113	0.9222
Extension at Break Load (mm); Y2	A-CS	6.41	17.10	0.0257
	B-SA	8.40	22.42	0.0179
	AB	0.0225	0.0600	0.8222
	A^2^	0.3200	0.8540	0.4236
	B^2^	0.2450	0.6538	0.4779
In Vitro Release after 30 min (%); Y3	A-CS	74.42	1.76	0.3395
	B-SA	14.61	0.3466	0.5974
	AB	9.18	0.2177	0.6726
	A^2^	35.14	0.8334	0.4286
	B^2^	40.49	0.9602	0.3994
In Vitro Release after 360 min (%); Y4	A-CS	272.67	6.47	0.0845
	B-SA	3.30	0.1263	0.7458
	AB	8.08	0.3092	0.6169
	A^2^	34.88	1.33	0.3317
	B^2^	133.35	5.10	0.1091

**Table 2:** Mechanical properties of different formulations of SSD biofilm.

Formulation	Average TS (Mpa)	Average EB (mm)	Average Thickness (mm)	Folding Endurance	Swelling Index (%)
F1	1.8 ± 0.041	3.2 ± 0.400	0.7 ± 0.007	11 ± 1.0414	2.312 ± 0.087
F2	5.4 ± 0.228	4.9 ± 0.410	0.9 ± 0.021	24 ± 1.412	3.712 ± 0.364
F3	0.4 ± 0.046	1.7 ± 0.082	0.9 ± 0.134	7.5 ± 0.707	3.002 ± 0.194
F4	5.2 ± 0.117	5.1 ± 0.483	0.6 ± 0	25.5 ± 0.709	3.307 ± 0.178
F5	2.5 ± 0.347	3.5 ± 0.683	0.6 ± 0.014	14.5 ± 1.414	2.911 ± 0.116
F6	0.7 ± 0.146	1.4 ± 0.093	0.4 ± 0.021	13 ± 0.709	3.547 ± 0.600
F7	2.2 ± 0.649	3 ± 0.687	0.6 ± 0.062	13.5 ± 1.412	3.936 ± 0.073
F8	4.5 ± 0.224	3.9 ± 0.765	0.6 ± 0.021	17 ± 2.121	2.165 ± 0.566
F9	0.6 ± 0.022	1.2 ± 1.722	0.6 ± 0.035	13.5 ± 1.414	3.768 ± 0.419

**Table 3:** Release kinetics model for SSD from different biofilm formulations.

Formula	Zero-Order Model		First-Order Model		Higuchi Diffusion Model		Korsmeyer–Peppas Model	
	r	Slope	r	Slope	r	Slope	r	*n*
F1	0.962	10.02	0.98	−0.21	0.992	31.22	0.996	0.37
F2	0.96	14.09	0.99	−0.24	0.992	36.46	0.995	0.39
F3	0.973	10.79	0.97	−0.28	0.992	32.74	0.995	0.31
F4	0.91	11.19	0.91	−0.19	0.965	35.3	0.985	0.393
F5	0.96	11.77	0.84	−0.20	0.991	36.41	0.98	0.429
F6	0.954	11.28	0.968	−0.19	0.986	34.62	0.969	0.580
F7	0.971	11.46	0.99	−0.2	0.994	34.94	0.995	0.354
F8	0.946	9.457	0.98	−0.1	0.981	29.2	0.982	0.387
F9	0.974	12.84	0.979	−0.12	0.992	38.33	0.987	0.831

**Table 4:** Independent factors and formulation matrix of SSD biofilm.

Independent Factors				Low Level (−1)		Middle (0)		High Level (+1)	
X1: Chitosan%				0.5		0.75		1	
X2: Sodium Alginate%				0.25		0.5		0.75	
Film composition	F1	F2	F3	F4	F5	F6	F7	F8	F9
SSD	1%								
Chitosan	0.5	0.75	0.75	1	0.75	0.5	1	1	0.5
Sodium Alginate	0.75	0.75	0.25	0.75	0.5	0.25	0.25	0.5	0.5
Ammonia solution	1 mL								
Propylene Glycol %	5%								

**Table 5:** Histological score used.

Score	Criteria
1–3	No to minimal cell accumulation. No granulation tissue or epithelial travel.
4–6	Thin, immature granulation that is dominated by inflammatory cells but has few fibroblasts, capillaries or collagen deposition. Minimal epithelial migration.
7–9	Moderately thick granulation tissue can range from being dominated by inflammatory cells to more fibroblasts and collagen deposition. Extensive neovascularization. Epithelium can range from minimal to moderate migration.
10–12	Thick, vascular granulation tissue dominated by fibroblasts and extensive collagen deposition. Epithelium partially to completely covering the wound.

## Data Availability

All the data are available with the corresponding author.
